# Dichlorido(dipyrido[3,2-*a*:2′,3′-*c*]phenazine)manganese(II)

**DOI:** 10.1107/S1600536808043468

**Published:** 2009-01-08

**Authors:** Mao-Liang Xu, Shu-Bo Sun, Xiu-Ying Li, Guang-Bo Che

**Affiliations:** a Xi’an Modern Chemistry Research Institute, Xi’an 710065, People’s Republic of China; b Department of Base, Shenyang Polytechnic College, Shenyang 110045, People’s Republic of China; c Department of Chemistry, Jilin Normal University, Siping 136000, People’s Republic of China

## Abstract

The complete mol­ecule of the title compound, [MnCl_2_(C_18_H_10_N_4_)_2_], is generated by crystallographic twofold symmetry with the Mn atom lying on the rotation axis. The Mn coordination geometry is a distorted *cis*-MnCl_2_N_4_ octa­hedron, arising from two *N*,*N*′-bidentate dipyrido[3,2-*a*:2′,3′-*c*]phenazine (DPPZ) ligands and two chloride ions. In the crystal structure, neighbouring mononuclear units pack together through π–π contacts between the DPPZ rings [shortest centroid–centroid distance = 3.480 (2) Å], leading to a chain-like structure along [001]. C—H⋯Cl hydrogen bonds complete the structure.

## Related literature

For background, see: Che *et al.* (2006 ▶, 2008 ▶); Xu *et al.* (2008 ▶).
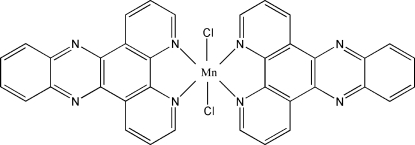


## Experimental

### 

#### Crystal data

[MnCl_2_(C_18_H_10_N_4_)_2_]
*M* *_r_* = 690.44
Monoclinic, 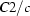

*a* = 8.4017 (17) Å
*b* = 12.256 (3) Å
*c* = 28.226 (6) Å
β = 95.09 (3)°
*V* = 2895.0 (10) Å^3^
*Z* = 4
Mo *K*α radiation
μ = 0.69 mm^−1^
*T* = 292 (2) K
0.38 × 0.24 × 0.21 mm

#### Data collection

Bruker SMART CCD area-detector diffractometer
Absorption correction: multi-scan (*SADABS*; Bruker, 2002 ▶) *T*_min_ = 0.821, *T*_max_ = 0.864
11873 measured reflections
2866 independent reflections
1748 reflections with *I* > 2σ(*I*)
*R*_int_ = 0.078

#### Refinement

*R*[*F* ^2^ > 2σ(*F* ^2^)] = 0.053
*wR*(*F* ^2^) = 0.120
*S* = 1.00
2866 reflections
243 parameters
H atoms treated by a mixture of independent and constrained refinement
Δρ_max_ = 0.32 e Å^−3^
Δρ_min_ = −0.43 e Å^−3^

### 

Data collection: *SMART* (Bruker, 2002 ▶); cell refinement: *SAINT* (Bruker, 2002 ▶); data reduction: *SAINT*; program(s) used to solve structure: *SHELXS97* (Sheldrick, 2008 ▶); program(s) used to refine structure: *SHELXL97* (Sheldrick, 2008 ▶); molecular graphics: *SHELXTL* (Sheldrick, 2008 ▶); software used to prepare material for publication: *SHELXTL*.

## Supplementary Material

Crystal structure: contains datablocks global, I. DOI: 10.1107/S1600536808043468/hb2884sup1.cif

Structure factors: contains datablocks I. DOI: 10.1107/S1600536808043468/hb2884Isup2.hkl

Additional supplementary materials:  crystallographic information; 3D view; checkCIF report

## Figures and Tables

**Table 1:** Selected bond lengths (Å)

Mn—N1	2.283 (3)
Mn—N2	2.316 (3)
Mn—Cl	2.4644 (12)

**Table 2:** Hydrogen-bond geometry (Å, °)

*D*—H⋯*A*	*D*—H	H⋯*A*	*D*⋯*A*	*D*—H⋯*A*
C2—H2⋯Cl^i^	1.09 (3)	2.67 (3)	3.737 (4)	168 (2)
C15—H15⋯Cl^ii^	1.04 (3)	2.64 (3)	3.648 (4)	163 (3)

Symmetry codes: (i) 
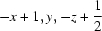
; (ii) 
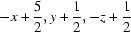
.

**Fig. 1.**
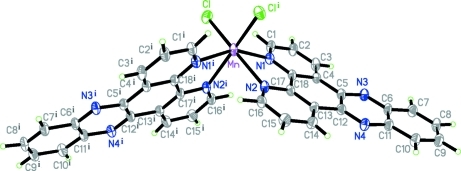
A view of (I). Displacement ellipsoids are drawn at the 30% probability level (arbitrary spheres for the H atoms). [Symmetry code: (i) -x + 2, y, -z + 1/2.]

**Fig. 2.**
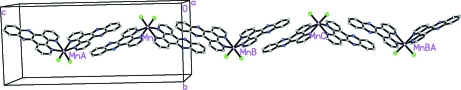
View of the supramolecular chain structure of (I) arising from π-π stacking. H atoms have been omitted. [Symmetry code: (A) -x + 2, y, -z + 1/2; (B) x, -y + 1, -z; (C) x, y, -z - 1; (BA) x, -y + 1, -z - 1.]

## supplementary crystallographic information

### Comment

1,10-Phenanthroline (phen) and its derivatives, as chelating N-containing aromatic ligands, has been extensively studied in the chemistry of coordination polymers (Che *et al.*, 2008). Here, we report the crystal structure of the title compound, [Mn(DPPZ)_2_Cl_2_] or [Mn(C_18_H_10_N_4_)_2_Cl_2_] (I), based on the dipyrido[3,2 - a:2',3'-c]-phenazine (DPPZ) ligand (Xu *et al.*, 2008).

In compound (I), the Mn atom (site symmetry 2) is coordinated in a distorted octahedral fashion (Fig. 1) by four N atoms from two DPPZ ligands and two Cl ions (Table 1). The DPPZ ring systems is almost planar and the dihedral angle between the two symmetry-related DPPZ planes is 70.66°.

π-π stacking interactions between the DPPZ ligands assemble mononuclear complex molecules into one-dimensional chains along (001) [centroid-centroid distances = 3.480 (2) Å] (Fig. 2). Finally, C—H···Cl hydrogen bonds involving the hydrogen of aromatic rings and the Cl ions further stabilize the crystal structure (Table 2).

### Experimental

The DPPZ ligand was synthesized according to the literature method of Che *et al.* (2006). A mixture of DPPZ, MnCl_2_ and water in a molar ratio of 2:1:5000 was sealed in a Teflon-lined autoclave and heated to 423 K for 3 d. Upon cooling and opening the bomb, yellow blocks of (I) were obtained (81% yield based on Mn).

### Refinement

The H atoms were located in a difference map and their positions were freely refined with a fixed U_iso_ value of 0.06Å^2^.

### Crystal data

**Table:**

[MnCl_2_(C_18_H_10_N_4_)_2_]	*F*(000) = 1404
*M**_r_* = 690.44	*D*_x_ = 1.584 Mg m^−^^3^
Monoclinic, *C*2/*c*	Mo *K*α radiation, λ = 0.71073 Å
Hall symbol: -C 2yc	Cell parameters from 2001 reflections
*a* = 8.4017 (17) Å	θ = 2.9–26.1°
*b* = 12.256 (3) Å	µ = 0.69 mm^−^^1^
*c* = 28.226 (6) Å	*T* = 292 K
β = 95.09 (3)°	Block, yellow
*V* = 2895.0 (10) Å^3^	0.38 × 0.24 × 0.21 mm
*Z* = 4	

### Data collection

**Table:**

Bruker SMART CCD area-detector diffractometer	2866 independent reflections
Radiation source: fine-focus sealed tube	1748 reflections with *I* > 2σ(*I*)
graphite	*R*_int_ = 0.078
ω scans	θ_max_ = 26.1°, θ_min_ = 2.9°
Absorption correction: multi-scan (*SADABS*; Bruker, 2002)	*h* = −10→10
*T*_min_ = 0.821, *T*_max_ = 0.864	*k* = −15→15
11873 measured reflections	*l* = −34→34

### Refinement

**Table:**

Refinement on *F*^2^	Primary atom site location: structure-invariant direct methods
Least-squares matrix: full	Secondary atom site location: difference Fourier map
*R*[*F*^2^ > 2σ(*F*^2^)] = 0.053	Hydrogen site location: inferred from neighbouring sites
*wR*(*F*^2^) = 0.120	H atoms treated by a mixture of independent and constrained refinement
*S* = 1.00	*w* = 1/[σ^2^(*F*_o_^2^) + (0.0518*P*)^2^] where *P* = (*F*_o_^2^ + 2*F*_c_^2^)/3
2866 reflections	(Δ/σ)_max_ = 0.001
243 parameters	Δρ_max_ = 0.32 e Å^−^^3^
0 restraints	Δρ_min_ = −0.42 e Å^−^^3^

### Special details

**Table:**

Geometry. All e.s.d.'s (except the e.s.d. in the dihedral angle between two l.s. planes) are estimated using the full covariance matrix. The cell e.s.d.'s are taken into account individually in the estimation of e.s.d.'s in distances, angles and torsion angles; correlations between e.s.d.'s in cell parameters are only used when they are defined by crystal symmetry. An approximate (isotropic) treatment of cell e.s.d.'s is used for estimating e.s.d.'s involving l.s. planes.
Refinement. Refinement of *F*^2^ against ALL reflections. The weighted *R*-factor *wR* and goodness of fit *S* are based on *F*^2^, conventional *R*-factors *R* are based on *F*, with *F* set to zero for negative *F*^2^. The threshold expression of *F*^2^ > σ(*F*^2^) is used only for calculating *R*-factors(gt) *etc*. and is not relevant to the choice of reflections for refinement. *R*-factors based on *F*^2^ are statistically about twice as large as those based on *F*, and *R*- factors based on ALL data will be even larger.

### Fractional atomic coordinates and isotropic or equivalent isotropic displacement parameters (Å^2^)

**Table:**

	*x*	*y*	*z*	*U*_iso_*/*U*_eq_	
C1	0.6671 (4)	0.3405 (3)	0.29912 (12)	0.0408 (9)	
C2	0.5622 (4)	0.3550 (3)	0.33399 (12)	0.0459 (10)	
C3	0.6118 (4)	0.4167 (3)	0.37276 (13)	0.0461 (10)	
C4	0.7619 (4)	0.4666 (3)	0.37635 (11)	0.0343 (8)	
C5	0.8223 (4)	0.5307 (3)	0.41737 (11)	0.0367 (9)	
C7	0.7118 (5)	0.6056 (3)	0.53324 (14)	0.0508 (11)	
C8	0.7738 (5)	0.6604 (3)	0.57158 (13)	0.0521 (11)	
C9	0.9253 (5)	0.7097 (3)	0.57276 (13)	0.0501 (11)	
C10	1.0139 (5)	0.7010 (3)	0.53419 (13)	0.0508 (11)	
C11	0.9520 (4)	0.6445 (3)	0.49315 (12)	0.0424 (9)	
C12	0.9766 (4)	0.5811 (3)	0.41822 (12)	0.0373 (9)	
C13	1.0710 (4)	0.5678 (3)	0.37731 (11)	0.0364 (9)	
C14	1.2220 (4)	0.6152 (3)	0.37604 (13)	0.0448 (10)	
C15	1.3054 (4)	0.5992 (3)	0.33699 (13)	0.0472 (10)	
C16	1.2389 (4)	0.5345 (3)	0.30016 (13)	0.0419 (9)	
C17	1.0135 (4)	0.5042 (3)	0.33907 (11)	0.0345 (8)	
C18	0.8568 (4)	0.4510 (3)	0.33849 (11)	0.0333 (8)	
C6	0.7985 (4)	0.5942 (3)	0.49265 (12)	0.0404 (9)	
N1	0.8113 (3)	0.3861 (2)	0.30102 (9)	0.0377 (7)	
N2	1.0963 (3)	0.4861 (2)	0.30080 (9)	0.0380 (7)	
N3	0.7343 (3)	0.5382 (2)	0.45415 (9)	0.0409 (8)	
N4	1.0403 (3)	0.6370 (2)	0.45531 (10)	0.0427 (8)	
Mn	1.0000	0.34811 (7)	0.2500	0.0383 (3)	
Cl	0.82991 (11)	0.22033 (8)	0.20077 (3)	0.0501 (3)	
H1	0.634 (4)	0.289 (3)	0.2722 (13)	0.060*	
H2	0.448 (4)	0.313 (3)	0.3296 (12)	0.060*	
H3	0.550 (4)	0.427 (3)	0.3977 (13)	0.060*	
H7	0.613 (4)	0.566 (3)	0.5315 (12)	0.060*	
H8	0.720 (4)	0.663 (3)	0.5996 (13)	0.060*	
H9	0.973 (4)	0.751 (3)	0.6018 (14)	0.060*	
H10	1.116 (4)	0.738 (3)	0.5341 (13)	0.060*	
H14	1.264 (4)	0.661 (3)	0.3997 (13)	0.060*	
H15	1.417 (4)	0.634 (3)	0.3341 (12)	0.060*	
H16	1.299 (4)	0.524 (3)	0.2703 (12)	0.060*	

### Atomic displacement parameters (Å^2^)

**Table:**

	*U*^11^	*U*^22^	*U*^33^	*U*^12^	*U*^13^	*U*^23^
C1	0.036 (2)	0.056 (3)	0.031 (2)	−0.0009 (19)	0.0073 (16)	−0.0062 (19)
C2	0.036 (2)	0.068 (3)	0.035 (2)	−0.003 (2)	0.0089 (17)	−0.006 (2)
C3	0.037 (2)	0.069 (3)	0.035 (2)	0.003 (2)	0.0195 (18)	0.000 (2)
C4	0.0330 (19)	0.043 (2)	0.0278 (19)	0.0012 (17)	0.0080 (15)	0.0006 (16)
C5	0.037 (2)	0.045 (2)	0.0288 (19)	0.0031 (18)	0.0086 (16)	0.0008 (17)
C7	0.058 (3)	0.061 (3)	0.036 (2)	0.005 (2)	0.019 (2)	−0.003 (2)
C8	0.061 (3)	0.069 (3)	0.029 (2)	0.004 (2)	0.0175 (19)	−0.008 (2)
C9	0.060 (3)	0.058 (3)	0.032 (2)	0.012 (2)	0.002 (2)	−0.008 (2)
C10	0.054 (3)	0.061 (3)	0.037 (2)	−0.002 (2)	0.007 (2)	−0.009 (2)
C11	0.049 (2)	0.050 (2)	0.029 (2)	0.005 (2)	0.0079 (17)	−0.0027 (18)
C12	0.041 (2)	0.042 (2)	0.030 (2)	0.0025 (18)	0.0095 (16)	−0.0019 (18)
C13	0.039 (2)	0.042 (2)	0.030 (2)	0.0012 (17)	0.0075 (16)	−0.0004 (17)
C14	0.045 (2)	0.051 (3)	0.039 (2)	−0.010 (2)	0.0098 (18)	−0.0088 (19)
C15	0.038 (2)	0.056 (3)	0.049 (2)	−0.009 (2)	0.0158 (19)	−0.002 (2)
C16	0.044 (2)	0.051 (2)	0.032 (2)	−0.0016 (19)	0.0128 (17)	0.0011 (19)
C17	0.0339 (19)	0.044 (2)	0.0269 (19)	0.0051 (17)	0.0078 (15)	0.0047 (17)
C18	0.0342 (19)	0.041 (2)	0.0257 (19)	0.0034 (17)	0.0065 (15)	0.0021 (17)
C6	0.047 (2)	0.050 (2)	0.025 (2)	0.0076 (19)	0.0090 (16)	−0.0031 (18)
N1	0.0344 (16)	0.049 (2)	0.0300 (17)	−0.0013 (15)	0.0053 (13)	−0.0018 (15)
N2	0.0379 (17)	0.050 (2)	0.0283 (16)	0.0001 (15)	0.0129 (13)	0.0004 (14)
N3	0.0396 (17)	0.054 (2)	0.0307 (17)	0.0027 (15)	0.0128 (14)	−0.0028 (15)
N4	0.0452 (18)	0.052 (2)	0.0317 (17)	0.0017 (15)	0.0091 (14)	−0.0061 (15)
Mn	0.0351 (4)	0.0540 (6)	0.0273 (4)	0.000	0.0113 (3)	0.000
Cl	0.0432 (6)	0.0631 (7)	0.0448 (6)	0.0003 (5)	0.0081 (4)	−0.0096 (5)

### Geometric parameters (Å, °)

**Table:**

C1—N1	1.330 (4)	C11—C6	1.429 (5)
C1—C2	1.390 (5)	C12—N4	1.323 (4)
C1—H1	1.01 (4)	C12—C13	1.467 (4)
C2—C3	1.365 (5)	C13—C17	1.383 (4)
C2—H2	1.08 (4)	C13—C14	1.399 (5)
C3—C4	1.397 (5)	C14—C15	1.372 (5)
C3—H3	0.92 (4)	C14—H14	0.92 (4)
C4—C18	1.402 (4)	C15—C16	1.385 (5)
C4—C5	1.453 (4)	C15—H15	1.04 (4)
C5—N3	1.330 (4)	C16—N2	1.339 (4)
C5—C12	1.434 (4)	C16—H16	1.03 (3)
C7—C8	1.339 (5)	C17—N2	1.354 (4)
C7—C6	1.418 (5)	C17—C18	1.468 (4)
C7—H7	0.96 (3)	C18—N1	1.351 (4)
C8—C9	1.406 (5)	C6—N3	1.356 (4)
C8—H8	0.95 (4)	Mn—N1	2.283 (3)
C9—C10	1.377 (5)	Mn—N2	2.316 (3)
C9—H9	1.02 (4)	Mn—Cl	2.4644 (12)
C10—C11	1.409 (5)	Mn—N1^i^	2.283 (3)
C10—H10	0.97 (4)	Mn—N2^i^	2.316 (3)
C11—N4	1.357 (4)	Mn—Cl^i^	2.4644 (12)
			
N1—C1—C2	123.4 (4)	C14—C15—C16	119.0 (3)
N1—C1—H1	119 (2)	C14—C15—H15	122 (2)
C2—C1—H1	118 (2)	C16—C15—H15	118.7 (19)
C3—C2—C1	118.1 (4)	N2—C16—C15	123.1 (3)
C3—C2—H2	123.9 (19)	N2—C16—H16	117.5 (19)
C1—C2—H2	118.0 (19)	C15—C16—H16	119 (2)
C2—C3—C4	120.7 (3)	N2—C17—C13	123.1 (3)
C2—C3—H3	123 (2)	N2—C17—C18	116.2 (3)
C4—C3—H3	117 (2)	C13—C17—C18	120.6 (3)
C3—C4—C18	117.1 (3)	N1—C18—C4	122.4 (3)
C3—C4—C5	122.9 (3)	N1—C18—C17	117.5 (3)
C18—C4—C5	119.9 (3)	C4—C18—C17	120.1 (3)
N3—C5—C12	121.5 (3)	N3—C6—C7	120.1 (3)
N3—C5—C4	118.7 (3)	N3—C6—C11	121.4 (3)
C12—C5—C4	119.8 (3)	C7—C6—C11	118.5 (3)
C8—C7—C6	120.8 (4)	C1—N1—C18	118.2 (3)
C8—C7—H7	125 (2)	C1—N1—Mn	124.7 (2)
C6—C7—H7	114 (2)	C18—N1—Mn	116.7 (2)
C7—C8—C9	121.4 (4)	C16—N2—C17	117.5 (3)
C7—C8—H8	121 (2)	C16—N2—Mn	125.4 (2)
C9—C8—H8	118 (2)	C17—N2—Mn	116.0 (2)
C10—C9—C8	120.0 (4)	C5—N3—C6	116.8 (3)
C10—C9—H9	118 (2)	C12—N4—C11	116.6 (3)
C8—C9—H9	122 (2)	N1^i^—Mn—N1	156.48 (15)
C9—C10—C11	120.1 (4)	N1^i^—Mn—N2	91.01 (10)
C9—C10—H10	120 (2)	N1—Mn—N2	71.63 (10)
C11—C10—H10	119 (2)	N1^i^—Mn—N2^i^	71.63 (10)
N4—C11—C10	119.5 (3)	N1—Mn—N2^i^	91.01 (10)
N4—C11—C6	121.3 (3)	N2—Mn—N2^i^	86.22 (14)
C10—C11—C6	119.2 (3)	N1^i^—Mn—Cl	100.04 (7)
N4—C12—C5	122.5 (3)	N1—Mn—Cl	94.86 (8)
N4—C12—C13	118.1 (3)	N2—Mn—Cl	165.09 (7)
C5—C12—C13	119.3 (3)	N2^i^—Mn—Cl	87.79 (8)
C17—C13—C14	118.0 (3)	N1^i^—Mn—Cl^i^	94.86 (8)
C17—C13—C12	120.1 (3)	N1—Mn—Cl^i^	100.04 (7)
C14—C13—C12	121.9 (3)	N2—Mn—Cl^i^	87.79 (8)
C15—C14—C13	119.4 (4)	N2^i^—Mn—Cl^i^	165.09 (7)
C15—C14—H14	119 (2)	Cl—Mn—Cl^i^	101.09 (6)
C13—C14—H14	122 (2)		

Symmetry codes: (i) −*x*+2, *y*, −*z*+1/2.

### Hydrogen-bond geometry (Å, °)

**Table:**

*D*—H···*A*	*D*—H	H···*A*	*D*···*A*	*D*—H···*A*
C2—H2···Cl^ii^	1.09 (3)	2.67 (3)	3.737 (4)	168 (2)
C15—H15···Cl^iii^	1.04 (3)	2.64 (3)	3.648 (4)	163 (3)

Symmetry codes: (ii) −*x*+1, *y*, −*z*+1/2; (iii) −*x*+5/2, *y*+1/2, −*z*+1/2.
